# Growth Mechanisms of Inductively-Coupled Plasma Torch Synthesized Silicon Nanowires and their associated photoluminescence properties

**DOI:** 10.1038/srep37598

**Published:** 2016-11-22

**Authors:** M. Agati, G. Amiard, V. Le Borgne, P. Castrucci, R. Dolbec, M. De Crescenzi, M. A. El Khakani, S. Boninelli

**Affiliations:** 1 Dipartimento di Fisica e Astronomia, Università di Catania, Via S. Sofia 64, I-95123 Catania, Italy; 2 CNR IMM-MATIS, Via S. Sofia 64, I-95123 Catania, Italy; 3 Institut national de la recherche scientifique, Centre-Énergie, Matériaux et Télécommunications, 1650 Blvd. Lionel Boulet, Varennes, QC, J3X 1S2, Canada; 4 Dipartimento di Fisica, Università di Roma “Tor Vergata”, Via della Ricerca Scientifica 1, Roma, 00133, Italy; 5 Tekna Plasma Systems Inc., 2935 Industrial Blvd., Sherbrooke, QC, J1L 2T9, Canada

## Abstract

Ultra-thin Silicon Nanowires (SiNWs) were produced by means of an industrial inductively-coupled plasma (ICP) based process. Two families of SiNWs have been identified, namely long SiNWs (up to 2–3 micron in length) and shorter ones (~100 nm). SiNWs were found to consist of a Si core (with diameter as thin as 2 nm) and a silica shell, of which the thickness varies from 5 to 20 nm. By combining advanced transmission electron microscopy (TEM) techniques, we demonstrate that the growth of the long SiNWs occurred via the Oxide Assisted Growth (OAG) mechanism, while the Vapor Liquid Solid (VLS) mechanism is responsible for the growth of shorter ones. Energy filtered TEM analyses revealed, in some cases, the existence of chapelet-like Si nanocrystals embedded in an otherwise silica nanowire. Such nanostructures are believed to result from the exposure of some OAG SiNWs to high temperatures prevailing inside the reactor. Finally, the intense photoluminescence (PL) of these ICP-grown SiNWs in the 620–950 nm spectral range is a clear indication of the occurrence of quantum confinement. Such a PL emission is in accordance with the TEM results which revealed that the size of nanostructures are indeed below the exciton Bohr radius of silicon.

One dimensional (1D) nanostructures have shown remarkable optical, electronic and chemical properties with respect to the bulk counterparts, because of the occurrence of both quantum confinement (QC) phenomena (related to the size-reduction) and surface effects (related to the increased aspect-ratio)^1^. For these reasons, a growing part of the scientific research has been devoted to the study of nanowires in recent years, both from a fundamental and a technological viewpoints. In particular, silicon nanowires (SiNWs) have been targeted as a highly attractive component for optoelectronic devices owing to the tunability of their optoelectronic properties through the control of their size^2^,^3^. Very recently hybrid dual layers constituted by randomly dispersed SiNWs embedded in conducting polymers have received great attention because these systems allow to achieve devices with enhanced electrical properties and compatible with plastics substrates^4^,^5^. Moreover, a recent work paper clarifies the details of how to achieve electrical contacts on randomly dispersed SiNW nanonet^6^.

To enable large scale production of such devices, the production of bulk quantities of SiNWs will constitute a major asset. In fact, this still remains a critical issue, since most of the techniques currently employed to synthesize SiNWs (such as chemical vapor deposition (CVD), molecular beam epitaxy (MBE), laser ablation or silicon monoxide evaporation) are not really scalable to produce large volumes and often result in low throughput.

In this article we report on the nanostructural investigation of randomly distibuted SiNWs produced by a novel and high throughput (of the order of hundreds of grams per day) inductively-coupled plasma (ICP) torch based process. The SiNWs investigated in the present work were formed as a by-product of an ICP spheroidization process exploited by Tekna Plasma Systems Inc. (Canada) to produce industrial quantities of micro spherical silicon^7^. Although the growth mechanisms of SiNWs have been well understood in the case of classical synthesis techniques (e.g. metal-catalysed MBE^8^, laser assisted growth^9^ and CVD^10^), the underlying mechanism through which one dimensional growth occurred in the ICP reactor has yet to be elucidated. Diverse physical mechanisms explaining the growth of SiNWs have been proposed in literature^11^. Among them, the Vapor-Liquid-Solid (VLS) mechanism was thouroughly described and widely recommended for the possibility to control the diameter and density of NWs^12^. In the VLS mechanism, metal nanoparticles are used as a catalyst to promote the NW growth, while the size of the metal nanoparticle dictates, to a large extent, its diameter. Although Au is the main metal catalyst used in VLS synthesis of SiNWs, Cu, Fe, Ni and Pt have been also reported^13^,^14^,^15^. On the other hand, the Oxide Assisted Growth (OAG) mechanism was also identified as a valid alternative to synthesize SiNWs without resorting to any metal catalysts^16^. In the OAG mechanism, instead of the metal catalyst, silicon suboxide clusters initiate the growth of the SiNWs. Indeed, the dangling bonds of the silicon suboxide clusters, present in the vapor phase, tend to form bonds with a silicon based substrate, becoming thereby a nucleation site for the subsequent growth of nanowires. Such a nucleated Si-suboxide cluster is fed by the adsorption of additional reactive Si suboxide clusters from the vapor, through the formation of Si-Si bonds^17^. On the other hand, the oxygen atoms in these piled silicon suboxide clusters are laterally expelled by the silicon atoms during the SiNW growth to the edges where they form a chemically inert SiO_2_ shell. Such a SiO_2_ sheath around the SiNW prevents its lateral growth and leaves the only possibility of the perpendicular growth of the Si nanowires^18^,^19^,^20^. Remarkably, among the advantages of the OAG based growth^21^, SiNWs were produced with diameters much thinner (i.e. minimum diameter 1.3 nm) than the VLS grown ones (minimum diameter 3 nm)^22^. Direct synthesis of SiNWs via the OAG growth mechanism has been reported in the cases of laser ablation^16^,^23^,^24^, thermal evaporation^23^,^25^ as well as CVD^26^ synthesis techniques.

An approach based on the detailed Transmission Electron Microscopy (TEM) analyses of the as-collected ICP-SiNWs was used in order to understand their growth mechanisms. We were thus able to demonstrate that the structural and chemical characterizations at the nanometric scale are key for the reconstruction of the mechanisms of their growth. The thorough characterizations of the morphology, structure and chemical composition of the ICP-SiNWs suggest that the growth occurs predominantly through the OAG mechanism. Statistically, only ~5% of the characterized ICP-SiNWs were found to grow from a metal catalyst nanoparticle present at their tip, according to the VLS mechanism. Furthermore, some OAG SiNWs exhibit an interesting inner structure, composed of well separated spherical Si nanocrystals (SiNCs) with a “chapelet-like” nanostructure or a string of “almond-shaped” SiNCs connected by a very thin SiNW, all embedded in a silica nanocylinder. In this case, the inner SiNW is very thin, with diameter as thin as ~2 nm, while the silica outer shell was found to have a thickness ranging from 4 to 6 nm.

Finally, the visible to near-infrared broad photoluminescence of these ICP-SiNWs is shown to correlate well with their TEM revealed nanostructural characteristics, supporting thereby the occurrence of quantum confinement in these silicon nanostructures.

## Results and Discussion

### Oxide Assisted Growth of SiNWs by Inductively Coupled Plasma

**Figure 1:**
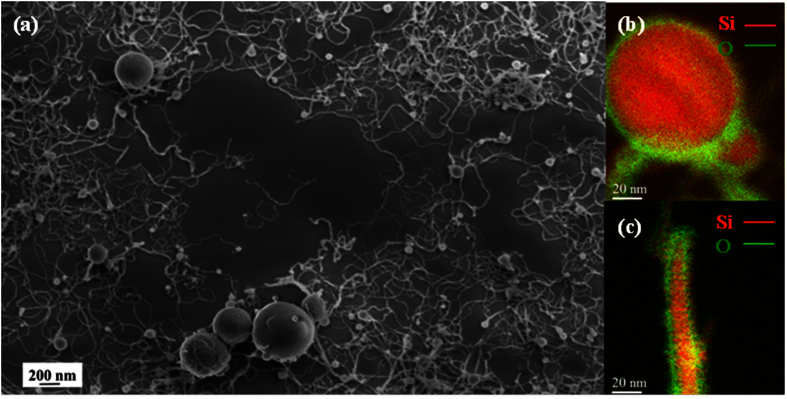
(**a**) Typical SEM image showing an ensemble of SiNSs and SiNWs. (**b**) EFTEM map (Si L_2,3_ edge at 99 eV in red and O K edge at 532 eV in green) of a typical core-shell Si/Si oxide NS; (**c**) EFTEM map of a core-shell Si/Si oxide NW.

**Figure 2:**
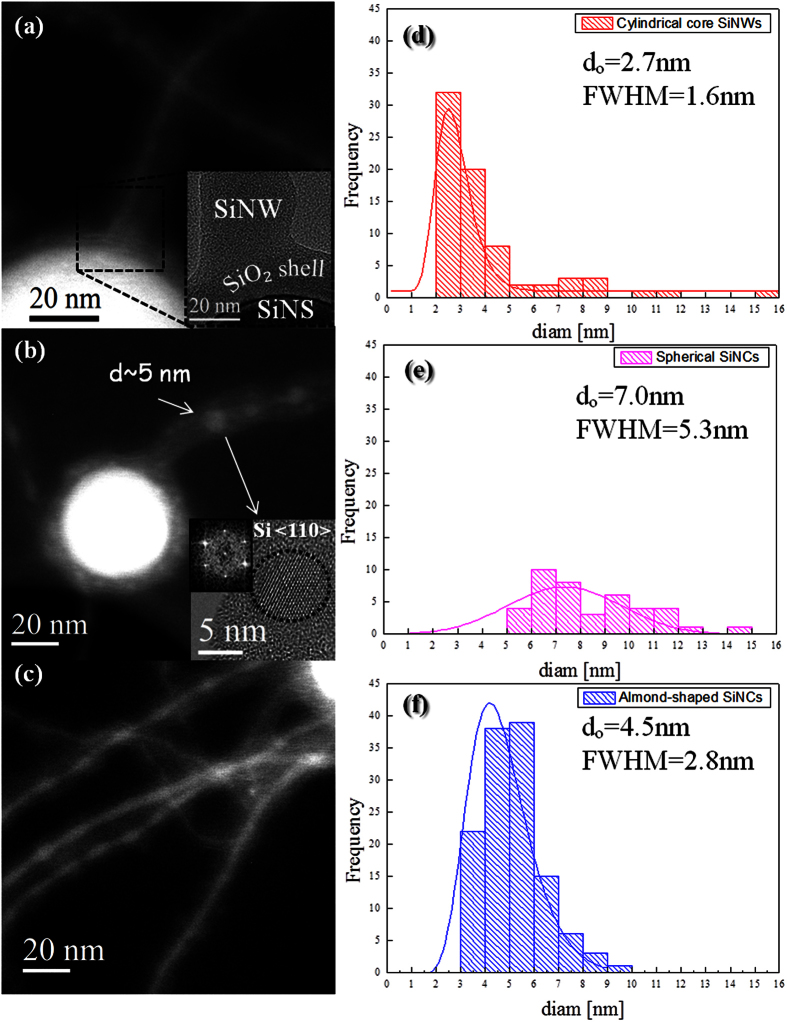
EFTEM acquired at 17 eV in correspondence of the Si plasmon energy loss, showing (**a**) continuous Si core, (**b**) spherical SiNCs whose crystalline nature is clarified by the HRTEM in the inset, showing a SiNC oriented in the <110> zone axis, and (**c**) almond-shaped SiNCs connected by a very thin SiNW. All these structures originate from the silica shell covering the SiNSs, as shown in the inset of (**a**). Statistical analyses to estimate the diameter distributions relative to: (**d**) the cylindrical SiNWs, (**e**) almond-shaped SiNCs and (**f**) spherical SiNCs forming the chapelet-like SiNWs.

In order to address the issue of SiNW growth in the ICP system, we firstly performed “large area” Scanning Electron Microscopy (SEM) observations. The typical SEM image reported in Fig. 1a depicts the as-collected Si-nanopowder material produced by the ICP system. We can clearly note a mixture of entangled, thin and very long (as long as 1–2 μm) SiNWs along with Si nanospheres (SiNSs) with sizes in the 50–500 nm range. On the other hand, Energy Filtered TEM (EFTEM) analyses, taken at 99 and 531 eV, corresponding to the Si L_2,3_ and O K edges, respectively, revealed that all the ICP-synthesized nanostructures are composed of a Si core (red colored) covered by a silicon oxide shell (green colored), as illustrated in Fig. 1b,c. The statistical analysis on several hundreds of nanostructures observed by EFTEM has allowed to determine that the diameter of the SiNS core is in the 45–480 nm range and they are surrounded by a ~8 ± 2 nm thick silicon oxide shell. Likewise, the core of the SiNWs is found to have diameters in the 2–15 nm range and is covered by a silica shell of which thickness varies from 4 to 15 nm. Further EFTEM examinations, performed at 17 eV, i.e. the plasmon energy losses of Si, revealed that the SiNW core presents an intriguing structure. Indeed, some SiNWs present a cylindrical Si nanocore wrapped by a concentric silica shell, as shown in Fig. 2a. We will refer to them as “cylindrical SiNWs”. Other SiNWs are found to consist of isolated spherical SiNCs encapsulated into a continuous silica cylinder, as shown in Fig. 2b. This configuration will be referred to as “chapelet-like” SiNW hereafter. Their crystalline nature is ascertained by High Resolution TEM (HRTEM) analyses, as shown in the inset of Fig. 2b, where a SiNC in the <110> zone axis is depicted. Figure 2c shows an intermediate case between the cylindrical SiNW and the chapelet-like configuration, where a modulated Si nanocore features a chain with “almond-shaped” SiNCs connected by a very thin Si wire (diameter of ~2 nm). These configurations will be referred to as “almond-shaped” SiNC chain hereafter. From the EFTEM images of Fig. 2a,b,c, it can be observed that the SiNWs directly develop from the silica shell covering the SiNSs, without any structural continuity with the SiNS core. The continuum silica shell covering the SiNS at the root of the growing NW is clearly depicted in the bright field TEM image reported in the inset of Fig. 2a, where the silica layer shows the typical contrast of the amorphous phase while the upper extremity of the SiNS appears dark owing to the diffraction contrast provoked by its crystalline phase.

**Figure 3:**
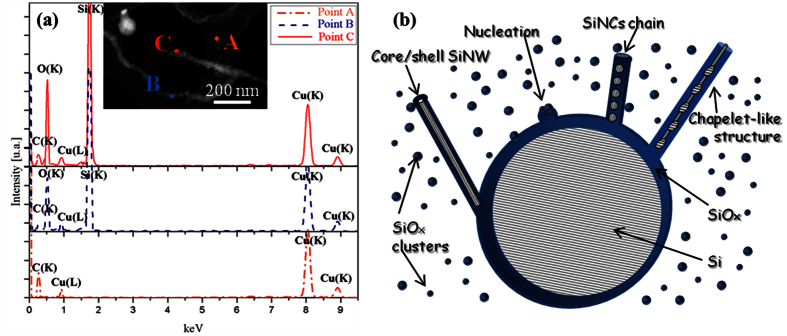
(**a**) Typical STEM-EDX spectra taken in correspondence of the carbon lacey TEM grid (A, orange color), along the SiNWs (B, blue color), on the top of the SiNWs (C, red color), as shown in the corresponding Dark Field STEM image. (**b**) Schematic of the OAG mechanism illustrating the nucleation operated by a Si suboxide cluster and the growth from a silica substrate. According to this mode, the formation of SiNC chains or chapelet-like nanostructures can also be possible.

This chain of almond-shaped SiNCs is rather intriguing but can be expected if one considers the Rayleigh instability affecting the cylindrical structure of the SiNWs exposed to high temperatures in the hot zone of the ICP chamber^27^. In fact, Rayleigh model predicts the morphological transformations of NWs when their shape becomes unstable under the influence of surface tension at elevated temperatures. Thus, it is highly likely that cylindrical SiNWs, subjected to high temperatures in the hot zone of the ICP reactor, tend to minimize their surface energy by evolving towards more-or-less spherical nanocrystals which can be still connected by a very thin central SiNW, eventually leading to the chapelet-like structures revealed by TEM. Similar Si nanosphere-chains have been also reported when Si nanowires were exposed to 1300 °C^28^. The TEM observations of hundreds of SiNWs allowed us to evaluate that statistically cylindrical SiNWs represent ~53% of the whole SiNWs present in the samples, while the “almond-shaped” SiNC chains count for ~35% and the “chapelet-like” NWs constitute ~12%. Moreover, from the size distributions and taking into account the accuracy of the TEM, we found that the cylindrical SiNW mean diameter is equal to 2.7 ± 1.0 nm, while the mean diameter of almond-shaped SiNCs is of 4.5 ± 1.4 nm and that of spherical SiNCs forming the chapelet-like chains is equal to 7.0 ± 2.7 nm, as reported in Fig. 2d,e,f, respectively. Moreover, further investigations on the chemical composition of the sample were carried out in order to better understand the mechanism through which the SiNW growth occurs in the ICP system. By means of Energy Dispersive X-Ray Spectroscopy in Scanning TEM (STEM)-EDX, we performed a chemical characterization at the nanometric scale on some tens of SiNWs and SiNSs, from which they sprout out. EDX spectra were acquired in correspondence of three different locations on each nanostructure, namely: (i) on the top of the NW (point C in the STEM image of Fig. 3a), (ii) along the wire (point B), (iii) above the carbon lacey Cu grid (point A, which is used as a background reference). For ~95% of the SiNWs examined, only the Si and O signals were observed, as shown by typical STEM-EDX spectra in Fig. 3a. This is in agreement with the previous EFTEM analyses, which put in evidence the core shell Si/silicon oxide structure. As the statistical STEM-EDX analyses attest that only Si and O are present in the sample, while no metal traces were detected along these wires, according to our EDX-system sensitivity (i.e. below 1 at.%), it can therefore be inferred that the OAG is the unique possible mechanism for the growth of these SiNWs^20^. We will further support this conclusion in the following paragraph where we will demonstrate that the small traces of Fe (0.2%) detected by inductively coupled plasma mass spectrometry (of which chemical sensitivity is much higher than EDX technique) are the responsible for the VLS growth of the remaining 5% of SiNW population.

**Figure 4:**
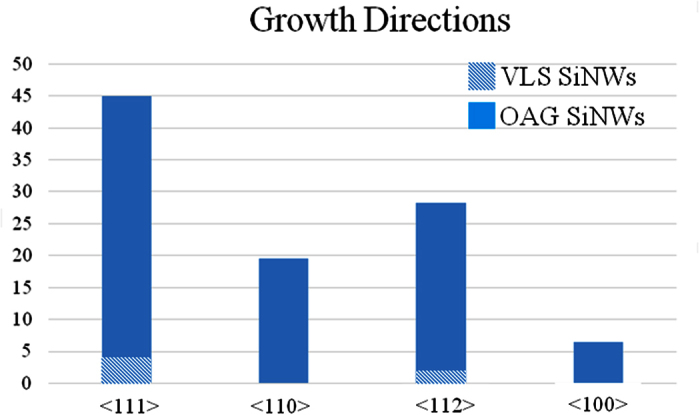
Statistical HRTEM analysis showing the percentage of the different growth directions.

In OAG mechanism, Si suboxide clusters induce the nucleation and growth of core-shell Si/silica NWs^19^. The occurrence of the OAG mechanism during the ICP synthesis can be delineated as follows. Inside the ICP reactor the Si feedstock is partially sublimated^29^ and the resulting highly reactive vapor actively interacts with oxygen present in the reactor. The oxygen originates mainly from the native oxide layer at the surface of Si feedstock powder which is partially released during the spheroidization process (it is commonly observed that powders processed by ICP exhibit enhanced purity as compared to feedstock^30^). Theoretical models have shown that hot vapors of Si and O are largely constituted of silicon suboxide clusters, which are highly reactive and tend to bond with other clusters^19^. Given the non-equilibrium conditions in the ICP reactor^29^, it is highly likely that the spheroidization occurs while some remaining hot vapors are present in the chamber. Concurrently, these SiNSs can act as substrate onto which other SiNWs can grow according to the OAG model, as schematically sketched in Fig. 3b (adapted from Fig. 5 in ref. 31). Indeed, if a Si suboxide cluster deposits on a SiNS, it can act as a nucleus that adsorbs additional reactive Si suboxide clusters from the vapor. Meanwhile, the oxygen atoms may diffuse to the edge. This results in the formation of a chemically inert Si oxide shell that prevents lateral growth, so only perpendicular growth is possible^19^,^20^. This OAG based unidirectional growth is initiated by the formation of a Si-Si bonding, established between the silicon suboxide cluster and the substrate^19^. This can be realized also if silicon oxide is used as a substrate, allowing the OAG based growth to start from a silica substrate, as observed in the case of our SiNWs in the inset of Fig. 2a and sketched in Fig. 3b. Moreover, the OAG mechanism is generally reported for synthesis temperatures between 850°–1100 °C^20^,^31^,^32^. Such temperatures are easily reachable in the post-discharge zone of the induction plasma torch. It can be thus inferred that the majority of the SiNWs investigated here are formed via the OAG mechanism in the post-discharge zone of the ICP reactor. It is noteworthy that the growth mechanism of SiNWs studied here follows the well estabished OAG mechanism^20^, although there are some specific features observed in the ICP grown SiNWs which have not been revealed before in OAG grown SiNWs. In particular, our ICP-SiNWs are found to grow on spherical surfaces, i.e. the SiNSs, which act as curved substrates. This being said, the different morphologies observed in these ICP-SiNWs are quite similar to those already reported by Peng *et al*.^28^ in OAG NWs grown via thermal evaporation. As discussed above, these three SiNW morphologies result from three different stages of surface tension minimization induced by the Rayleigh instability at different temperature zones. Interestingly, in the intermediate case, ultrathin ICP-SiNWs with a diameter as thin as 2 nm are produced and found to connect neighboring Si nanocrystals. On the other hand, HRTEM analyses have revealed that statistically ~45% of SiNWs have a crystalline core oriented along the <111> direction, while the 28% of them is found to develop along the <112> direction, as shown in Fig. 4. In addition, ~20% of our ICP-SiNWs grow along the <110> direction and ~7% have the <100> orientation. In literature, it has been demonstrated that the preferential growth direction for OAG NWs could be the <111> or <112>, depending on the different temperatures at which OAG takes place, while the <110> and <100> have also been observed in lower percentage. In fact, the <111> orientation is favored at higher temperatures^33^, while the <112> predominates at relatively lower temperatures^20^. Since in the ICP system, we are in presence of high temperatures regime (up to 10,000 K particulalry in the discharge-zone) followed by a rapid non-equilibrium thermal quenching, both growth directions (<111> and <112>) can occur with nonetheless a predominace for the high-temperatures promoted <111> orientation. Finally, we also demonstrated that the ICP process can produce OAG-SiNWs in the less favored growth directions (i.e. <110> and <100>) but in much lesser proportion, in agreement with literature^20^.

### Vapor-Liquid-Solid grown SiNWs by Inductively Coupled Plasma

**Figure 5:**
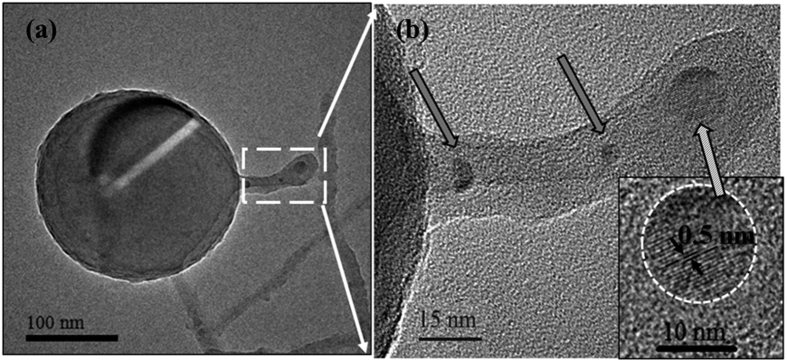
(**a**) Bright Field TEM image showing a SiNS, whose core generates three perpendicular NWs. (**b**) Enlarged image on the NWs. Arrows indicate three small nanoparticles at the end of each wire; in particular the top particle, enlarged in the inset, presents fringes corresponding to 0.5 nm interplanar distance.

**Figure 6:**
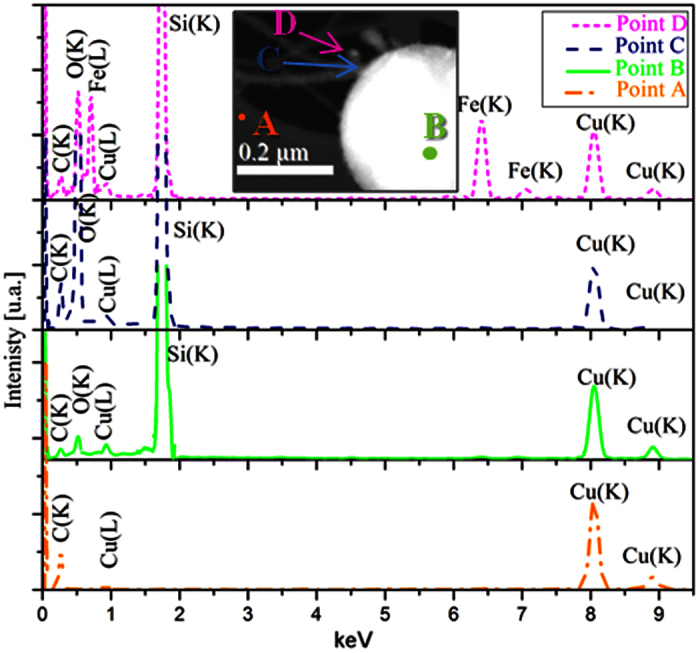
STEM-EDX spectra taken on the carbon lacey TEM grid (A, orange color), on the SiNS (B, green color), along the SiNW (C, blue color) and at the tip of the SiNW (D, magenta color), as shown in the Dark Field STEM image in the inset.

**Figure 7:**
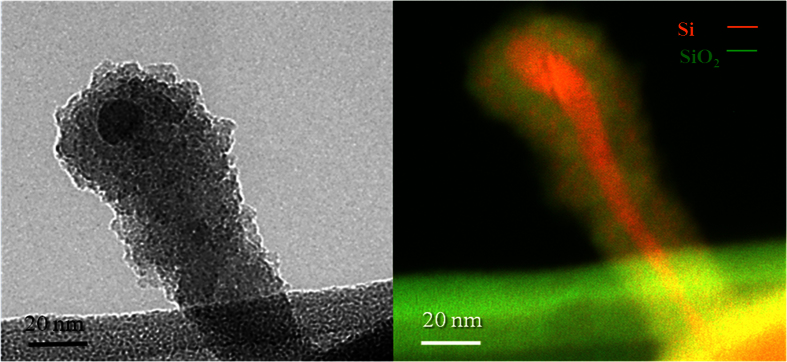
(**a**) Bright Field TEM of a SiNW having a Fe nanoparticle at the tip. (**b**) EFTEM image of a typical VLS NW (Si plasmon loss at 17 eV in red and SiO_2_ plasmon loss at 23 eV in green). The comparison with the corresponding Bright Field image demonstrates that the Si core evolves below the Fe particle.

About the 5% of the ICP-SiNWs were found to exhibit diverging characteristics as compared to the ones analyzed above. They are shorter, with a length rarely exceeding 150 nm. They show a dark spherical particle at their tip and are found to sprout out directly from the Si core of a massive underlying silicon nanosphere, as illustrated in Fig. 5a, growing preferentially along the <111> direction, as demonstrated in Fig. 4. A higher magnification view of the SiNW-SiNS interface (see Fig. 5b) shows three SiNWs superimposed on each other. Indeed, correspondingly, we can see three nanoparticles (indicated by the arrows) at the tips of these three SiNWs growing perpendicularly from the larger SiNS. On the other hand, the HRTEM image of the top particle, shown in the inset of Fig. 5b, presents a series of fringes separated by a distance equal to 0.5 nm. Such an interplanar distance is compatible with the 0.5 nm distance between (001) planes in β-FeSi_2_^15^. Moreover, the presence of such compound is in agreement with the Fe-Si phase diagram, which shows that FeSi_2_ is the most stable compound in the Si-rich zone of the diagram at high temperatures^15^, which is a condition met in the ICP discharge. To ascertain the presence of Fe-containing nanoparticles at the tip of these shorter SiNWs, STEM-EDX chemical analyses were performed at four different locations, namely: (A) on the carbon lacey Cu grid (used as a background reference), (B) over the SiNS from which the SiNW originates, (C) along the nanowire and (D) above the small particle at the tip of the nanowire. Figure 6 shows these A, B, C and D locations along with their associated STEM-EDX spectra. On the SiNS (point B), only Si and O signals were detected, in addition of Cu and C signals originating from the TEM grid. Remarkably, the spectrum acquired from region D (magenta dotted line) clearly demonstrates the presence of Fe, while there is no evidence of any metal trace in the spectrum acquired along the SiNW (region C, blue dashed line). These observations corroborate well with the VLS model predictions, where metal-containing particles at the tip of the SiNWs are typical marks of the VLS tip-growth mechanism^15^. Several STEM-EDX analyses ensure that dark nanoparticles at the tip of the SiNWs, like the one shown in Fig. 7a, always contain Fe. Figure 7b illustrates the associated EFTEM of Fig. 7a, taken at 17 eV and 23 eV respectively, corresponding to the Si and SiO_2_ energy plasmon losses. By comparing these images, it can be noted that the catalyst nanoparticle size (of ~12 nm) matches exactly the SiNW core diameter at its tip. Moreover, it is worth noting that there is a unique Si core continuously connecting the NW and the NS from which the nanowire originated. This confirms that the SiNSs acted as a supporting substrate for the SiNW growth in the VLS growth mechanism. In addition, one can also notice that the diameter of the SiNW (Fig. 7b) at the top is thicker than at its basis, suggesting that the radial growth is enhanced at the top of NWs. We could envisage that this peculiar enlargement is due to the surface diffusion of Si atoms above the metal catalyst towards the solid-liquid interface, where they are finally incorporated into the solid phase^11^. Since Wang and coworkers^11^ demonstrated that surface diffusion is enhanced at lower temperatures we can conjecture that this surface diffusion mechanism becomes competitive with respect to the axial growth during the cooling phase of the ICP process.

Finally, it should be noticed that the Fe concentration in the as collected powder is ~1770 ppm (i.e. less than the 0.2%) as measured by inductively coupled plasma mass spectrometry. This very low Fe concentration is not sufficient to sustain the growth of the whole NW population but it was found to be the responsible for the VLS growth of about the 5% of the whole population of NWs.

These results clearly demonstrate that the formation of SiNWs through the catalyzed VLS growth is also possible in the ICP reactor as long as a metal catalyst is present in the reaction zone. In the present experiment, even if the catalyzed SiNWs growth was not intended, the Si feedstock used in the spheroidization process contained some Fe impurities. Therefore, we argue that some Fe nanoparticles form on the larger SiNSs. These Fe nanoparticles become supersaturated by diffused Si atoms, leading thereby to the precipitation of Si and its crystallization as a nanowire. Further reaction with the oxygen causes the oxidation of the structures inducing the oxide outer-shell formation. Therefore, VLS growth mechanism cannot be ruled out provided that sufficient metal catalyst is available during the ICP synthesis process.

Finally, we can notice that VLS-SiNWs synthetized by ICP process are shorter than the OAG ones, indicating a faster growth rate for these latter. It should be considered that the main factor influencing the VLS kinetics is the Gibbs-Thomson effect which establishes a dependence of the growth rate on the SiNW diameter. Indeed, the growth rate decreases for those SiNWs having smaller diameter^34^. On the other hand, the growth rate by OAG mechanism mainly depends on the concentration of Si atoms in the silicon suboxide clusters available in the gas phase, i.e. clusters richer in Si more probably form Si-Si bonds and promote the SiNW growth^11^. Thus, in this case the growth rate does not depend on the NW diameter.

The coexistence of ultra-long OAG and VLS SiNWs has been reported previously, where no significant difference in terms of their growth rates has been noticed^16^,^32^. Nevertheless, it should be considered that in these papers no VLS SiNWs having a diameter smaller than 50 nm were observed; if thinner NWs were present, their growth would have been slower than the OAG ones, owing to the Gibbs-Thomson effect^32^. In our ICP experiment, VLS-SiNWs have diameters not greater than 20 nm; hence, we can conclude that the kinetics of their growth is significantly retarded respect to OAG because of the Gibbs-Thomson effect.

### Correlation between optical and structural properties

**Figure 8:**
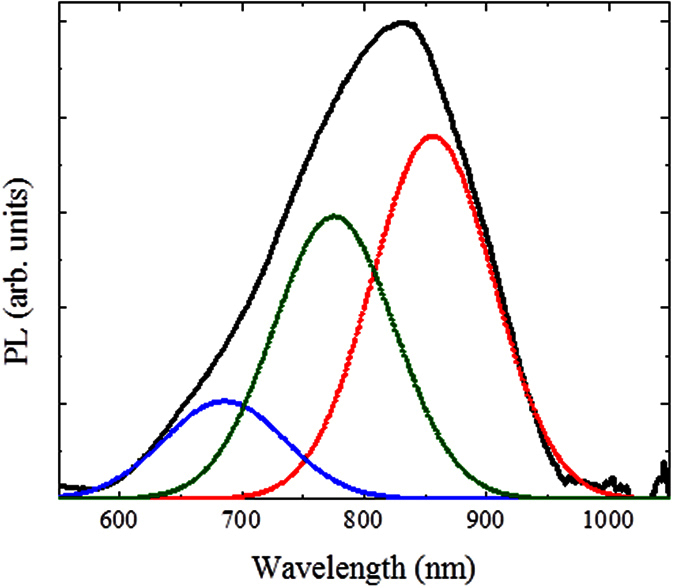
Typical room temperature PL spectrum of the as collected ICP-SiNWs following their excitation with a 405 nm laser line.

Once the TEM analyses confirmed the nanosize of the ICP-SiNWs, we have investigated their PL properties to check the possible occurrence of QC effects in these Si nanostructures. Figure 8 shows a typical PL spectrum of the ICP-SiNWs following their excitation with a 405 nm laser beam at room temperature. This PL emission is seen to be intense and centered around 830 nm with a full width at half maximum (FWHM) of ~185 nm. This PL in the visible-infrared range (620–950 nm) from our silicon nanostructures is a clear indication of the occurrence of QC (keeping in mind that Si is an indirect bandgap material at ~1100 nm). One can also notice that this broad PL spectrum is asymmetric and contains apparent shoulders, suggesting its deconvolution into different components. In fact, the PL spectrum can be fitted with three components having a FWHM of ~120 nm (this line width is close to that of ~100 nm which was reported for monodisperse silicon nanoparticles^35^). Thus, the three PL components are located at ~685 nm, ~775 nm and ~855 nm, most presumably associated with light emission from electron-hole recombination into quantum confined Si nanostructures having three different sizes and morphologies, namely, SiNWs, almond-shaped SiNCs and spherical SiNCs. Indeed, if we distinguish 1D (the cylindrical SiNWs) and 0D (spherical and almond-shaped SiNCs) nanostructures, we can correlate their mean size with the corresponding PL emission. In the former case, the theoretical curve for the predicted bandgap of cylindrical SiNWs as a function of the different growth directions has been studied by Zhao *et al*.^36^. A bandgap of about 1.75 eV (~700 nm) is expected for cylindrical SiNWs with ~2.5 nm-diameter, growing along the <112> direction (this growth direction constitutes ~30% of our ICP-SiNWs, as shown in Fig. 4). Considering that the mean diameter of our cylindrical ICP-SiNW is equal to 2.7 ± 1.0 nm, we can expect that most of them contribute to the high-energy component (around 685 nm) of the observed PL spectrum of Fig. 8. In the case of 0D nanostructures, calculations were performed by approximating the almond-shaped SiNCs to spherical SiNCs having the same diameter. Thus, the size of the silicon nanocrystals can be connected with their bandgap *E*_*PL*_ (or PL emission line) according to the following relationship^37^:


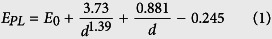


where *E*_*0*_ is the bandgap of bulk Si (*E*_*0*_ = 1.17 *eV*) and *d* is the SiNC diameter. An emission line at 775 nm (~1.60 eV) is expected for SiNCs having mean size ~3.2 nm, indicating that the almond-shaped SiNCs can also contribute to the second component of the total PL spectrum. According to Eq. (1), the low energy component located at 855 nm (1.45 eV) corresponds to SiNCs mean size ~3.8 nm. This latter PL component is believed to be due to almond-shaped SiNCs having a size close to 4 nm. Hence, spherical SiNCs do not contribute to the PL signal since their size (≥5 nm) is larger than the exciton Bohr radius in Si (~4.5 nm)^38^.

In summary, we have achieved systematic structural morphological and optoelectronic studies of ICP produced Si nanostructures, throughout an approach based on the combination of Electron Microscopy techniques (SEM, HRTEM, EF-TEM and STEM-EDX) and photoluminescence. The as-collected Si nanomaterial was found to be a mixture of SiNWs and SiNSs, which were shown to consist of a Si nano-core covered by a silica shell. The TEM based investigations revealed that the vast majority (~95%) of the ICP produced SiNWs grew according to the OAG mechanism, as no catalyst was intentionally introduced in the feedstock. Indeed, these OAG grown SiNWs were found to grow directly onto larger SiNSs and have diameters of ~2–15 nm and lengths as long as 2 μm. Some of these OAG synthesized SiNWs were found to present an intriguing internal nanostructure, made of chapelet-like Si nanocrystals, eventually connected by an extremely thin SiNW and embedded into an otherwise continuous silica nanocylinder. This is believed to result from Rayleigh instability due to rapid post-synthesis heating of the SiNWs before their cooling down in the final part of the reactor. Such Si nanostructures provide a new kind of nanocomposite, where QC effects occur. Indeed, our PL studies confirmed the occurrence of QC in these Si nanostructures as they exhibited an intense and broad PL emission over all the 620–950 nm spectral range. In fact, three PL components have been isolated and associated with three different shapes and sizes of the ICP grown Si nanostructures. Our investigations also demonstrated that the VLS growth is also possible in the ICP synthesis, given that an appropriate catalyst is used along with the silicon feedstock. In fact, with some Fe impurities present inside the ICP reaction zone, we have found almost 5% of the SiNWs to be grown through the VLS mechanism. These VLS-grown SiNWs exhibited different characteristics, as they are shorter (no longer than 150 nm), grow from the Si core of the larger SiNSs and present a Fe-rich nanoparticle at their tip. We conclude that the formation of SiNSs is mainly due to the spheroidization process inside the plasma chamber, while the SiNWs can grow according to both the OAG and VLS mechanisms (with a predominance of the OAG mechanism if no intentional metal catalyst is added, as is the case here). These results constitute a solid basis towards a better-controlled growth of SiNWs via the ICP technique, where the content and nature of the catalyst can be controlled^39^. The ICP is a genuinely bulk process, which can be advantageously exploited for large scale production of thin SiNWs needed to integrate Si into attractive large-area optoelectronic devices and flexible electronics.

## Methods

### Materials Synthesis

The SiNWs studied here were formed as a by-product of the ICP-based spheroidization process used to produce large volumes of silicon microspheres from a silicon powder feedstock^7^. In this process, the pure Si powder feedstock (average particle size of 115 μm and 99.5% purity) is introduced in the Ar/H_2_ plasma torch system with Ar as carrier gas. Most of the feedstock powder is melted in-flight and minimizes its surface energy by forming Si spheres of equivalent mean diameter (e.g. 115 μm) that are collected in the first collector of the ICP reactor. Concomitant to the in-flight melting of the silicon particles in the hot zone of the ICP torch, a fraction of the Si powder sublimates and the resulting Si vapor condenses in the lower temperature zone of the reactor, leading to the formation of lighter Si nanopowders. These latter are carried downstream with the torch gases and are retained in the second collector. This Si-nanopowder is referred to as the “as-collected” powder and has been thoroughly analysed in the present study.

### Nanostructural and Optical Characterization

Structural and chemical characterizations were performed using a Field Emission SEM Zeiss Supra 25, a JEOL JEM 2010 F EFTEM operating at an acceleration voltage of 200 kV and a spherical aberration corrected STEM ARM JEOL, operating at 100 kV and equipped with a large area (100 mm^2^) Energy Dispersive X-Ray spectrometer (EDX). The as-collected powder was dissolved into isopropyl alcohol and sonicated for 5 min. Few drops of the resulting solution were dispersed on a Si substrate and on carbon lacey Cu or Au grids, for SEM and TEM analyses, respectively. The PL measurements were performed with a 405 nm (12 mW) solid state laser excitation line at room temperature. The emitted light was collected through an optical fiber into a USB5000 Ocean optics CCD spectrometer, with a 405 nm notch filter placed between the laser and the sample and a 475 nm long pass filter placed between the sample and the spectrometer. All PL spectra were corrected for the system response curve.

## Additional Information

**How to cite this article**: Agati, M. *et al*. Growth Mechanisms of Inductively-Coupled Plasma Torch Synthesized Silicon Nanowires and their associated photoluminescence properties. *Sci. Rep.* **6**, 37598; doi: 10.1038/srep37598 (2016).

**Publisher's note:** Springer Nature remains neutral with regard to jurisdictional claims in published maps and institutional affiliations.

## References

[b1] Xia Y. N. . One-dimensional nanostructures: Synthesis, characterization, and applications. Adv. Mater. 15, 353–389 (2003).

[b2] Holmes J., Johnston K., Doty R. & Korgel B. Control of thickness and orientation of solution-grown silicon nanowires. Science 287, 1471–1473 (2000). 10688792 10.1126/science.287.5457.1471

[b3] Irrera A. . Quantum confinement and electroluminescence in ultrathin silicon nanowires fabricated by a maskless etching technique. Nanotechnology 23, 075204, 10.1088/0957–4484/23/7/075204 (2012). 22273546

[b4] Huang B.-R. . Silicon nanowire networks for the application of field effect phototransistor, Mater. Sc. and Eng. C. 27, 1197–1200 (2007).

[b5] Hsieh G.-W. . Dual layer semiconducting nanocomposite of silicon nanowire and polythiophene for organic-based field effect transistors. Organic Electronics 35, 158–163 (2016).

[b6] Ternon C. . Silicon Nanowires: Low Temperature Processing to Form Oxidation Insensitive Electrical Contact at Silicon Nanowire/Nanowire Junctions. Adv. Electron. Mater. 1, 1500172, 10.1002/aelm.201500172 (2015).

[b7] Guo J. Y., Gitzhofer F. & Boulos M. I. Induction plasma synthesis of ultrafine SiC powders from silicon and CH4. J. Mater. Sci. 30, 5589–5599 (1995).

[b8] Schubert L. . Silicon nanowhiskers grown on<111> Si substrates by molecular-beam epitaxy. Appl. Phys. Lett. 84, 4968–4970 (2004).

[b9] Yang Y. H., Wu S. J., Chin H. S., Lin P. I. & Chen Y. T. Catalytic growth of silicon nanowires assisted by laser ablation. J. Phys. Chem. B 108, 846–852 (2004).

[b10] Hofmann S. . Gold catalyzed growth of silicon nanowires by plasma enhanced chemical vapor deposition. J. Appl. Phys. 94, 6005–6012 (2003).

[b11] Wang N., Cai Y. & Zhang R. Q. Growth of nanowires. Mater. Sci. Eng. R-Rep. 60, 1–51 (2008).

[b12] Cui Y., Lauhon L. J., Gudiksen M. S. & Wang J. Diameter-controlled synthesis of single-crystal silicon nanowires. Appl. Phys. Lett. 78, 2214–2216 (2001).

[b13] Wagner R. S. & Ellis W. C. Vapor-liquid-solid mechanism of single crystal growth. Appl. Phys. Lett. 4, 89–90 (1964).

[b14] Arbiol J., Kalache B., Roca P., Morante J. R. & Fontcuberta A. Influence of Cu as a catalyst on the properties of silicon nanowires synthesized by the vapour – solid – solid mechanism. Nanotechnology 18, 10.1088/0957-4484/18/30/305606 (2007).

[b15] Morales A. M. & Lieber C. M. A Laser Ablation Method for the Synthesis of Crystalline Semiconductor Nanowires. Science 279, 7–10 (1998). 10.1126/science.279.5348.208 9422689

[b16] Zhang Y. F. . Silicon nanowires prepared by laser ablation at high temperature. Appl. Phys. Lett. 1835, 1835–1837 (1998).

[b17] Zhang R. Q., Chu T. S., Cheung H. F., Wang N. & Lee S. T. High reactivity of silicon suboxide clusters. Phys. Rev. B 64, 113304, 10.1103/PhysRevB.64.113304 (2001).

[b18] Zhang R. Q. & Fan W. J. Structures and properties of silicon oxide clusters by theoretical investigations. J. Clust. Sci. 17, 541–563 (2006).

[b19] Zhang R., Chu T. & Cheung H. Mechanism of oxide-assisted nucleation and growth of silicon nanostructures. Mater. Sci. Eng. C 16, 31–35 (2001).

[b20] Zhang R. Q., Lifshitz Y. & Lee S. T. Oxide-Assisted Growth of Semiconducting Nanowires. Adv. Mater. 15, 635–640 (2003).

[b21] Ma D. D. D., Lee C. S., Au F. C. K., Tong S. Y. & Lee S. T. Small-diameter silicon nanowire surfaces. Science 299, 1874–7 (2003). 12595610 10.1126/science.1080313

[b22] Wu Y. . Controlled growth and structures of molecular-scale silicon nanowires. Nano Lett. 4, 433–436 (2004).

[b23] Wang N., Tang Y. F., Zhang C. S. Lee & Lee S. T. Lee. Nucleation and growth of Si nanowires from silicon oxide. Phys. Rev. B 58, 16024–16026 (1998).

[b24] Wang N. . Transmission electron microscopy evidence of the defect structure in Si nanowires synthesized by laser ablation. Chem. Phys. Lett. 283, 368–372 (1998).

[b25] Wang N. . Si nanowires grown from silicon oxide. Chem. Phys. Lett. 299, 237–242 (1999).

[b26] Yao Y., Li F. & Lee S. T. Oriented silicon nanowires on silicon substrates from oxide-assisted growth and gold catalysts. Chem. Phys. Lett. 406, 381–385 (2005).

[b27] Rayleigh L. On the instability of jets. Proc. London Math. Soc. 10, 4–13 (1879).

[b28] Peng H. Y. . Bulk-quantity Si nanosphere chains prepared from semi-infinite length Si nanowires. J. Appl. Phys. 89, 727–731 (2001).

[b29] Jiayin G. . Development of Nanopowder Synthesis Using Induction Plasma. Plasma Sci. Technol. 12, 188–199 (2010).

[b30] *Nanopowders* Available at: www.tekna.com, (Accessed: 17/06/2016) (2016).

[b31] Lee S. T., Wang N. & Lee C. S. Semiconductor nanowires: synthesis, structure and properties. Mater. Sci. Eng. A 286, 16–23 (2000).

[b32] Peng H. Y. . Temperature Dependence of Si Nanowire. Adv. Mater. 13, 317–320 (2001).

[b33] Gole J. L. . Direct synthesis of silicon nanowires, silica nanospheres, and wire-like nanosphere agglomerates, Appl. Phys. Lett. 76, 2346–2348 (2000).

[b34] Givargizov E. I. Fundamental aspects of VLS growth. J. Cryst. Growth 31, 20–30 (1975).

[b35] Kang Z. . A polyoxometalate-assisted electrochemical method for silicon nanostructures preparation: From quantum dots to nanowires. J. Am. Chem. Soc. 129, 5326–5327 (2007). 17407292 10.1021/ja068894w

[b36] Zhao X., Wei C. M., Yang L. & Chou M. Y. Quantum confinement and electronic properties of silicon nanowires. Phys. Rev. Lett. 92, 236805, 10.1103/PhysRevLett.92.236805 (2004). 15245186

[b37] Ledoux G. . Photoluminescence properties of silicon nanocrystals as a function of their size. Phys. Rev. B 62, 15942–15951 (2000).

[b38] Barbagiovanni E. G., Lockwood D. J., Simpson P. J. & Goncharova L. V. Quantum confinement in Si and Ge nanostructures. J. Appl. Phys. 111, 034307, 10.1063/1.2179147 (2012).

[b39] Lamontagne P. . Synthesis of silicon nanowires from carbothermic reduction of silica fume in RF thermal plasma. Phys. Status Solidi a-Applications Mater. Sci. 211, 1610–1616 (2014).

